# Endo-S-c-di-GMP Analogues-Polymorphism and Binding Studies with Class I Riboswitch

**DOI:** 10.3390/molecules171113376

**Published:** 2012-11-09

**Authors:** Jie Zhou, David A. Sayre, Jingxin Wang, Nirmal Pahadi, Herman O. Sintim

**Affiliations:** Department of Chemistry and Biochemistry, University of Maryland, College Park, MD 20742, USA

**Keywords:** c-di-GMP, endo-S-c-di-GMP, polymorphism, G-quadruplex, analogues, DOSY, Vc2 RNA, fluorescence, aptamer

## Abstract

C-di-GMP, a cyclic guanine dinucleotide, has been shown to regulate biofilm formation as well as virulence gene expression in a variety of bacteria. Analogues of c-di-GMP have the potential to be used as chemical probes to study c-di-GMP signaling and could even become drug leads for the development of anti-biofilm compounds. Herein we report the synthesis and biophysical studies of a series of c-di-GMP analogues, which have both phosphate and sugar moieties simultaneously modified (called endo-S-c-di-GMP analogues). We used computational methods to predict the relative orientation of the guanine nucleobases in c-di-GMP and analogues. DOSY NMR of the endo-S-c-di-GMP series showed that the polymorphism of c-di-GMP can be tuned with conservative modifications to the phosphate and sugar moieties (conformational steering). Binding studies with Vc2 RNA (a class I c-di-GMP riboswitch) revealed that conservative modifications to the phosphate and 2'-positions of c-di-GMP dramatically affected binding to class I riboswitch.

## 1. Introduction

**Figure 1:**
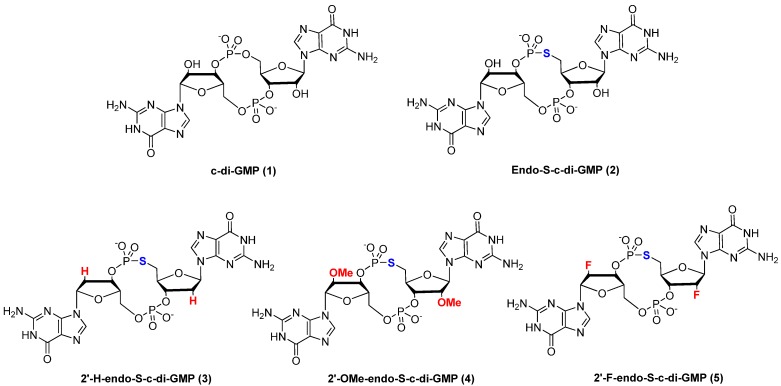
Family of dual-modified c-di-GMP analogues synthesized.

Cyclic diguanylic monophosphate (c-di-GMP) is a second messenger in bacteria and plays a central role in biofilm formation and the regulation of virulence-related factors in many bacteria [1]. C-di-GMP is produced from two guanosine triphosphate (GTP) molecules via diguanylate cyclases (DGCs), which contain the common protein domain GGDEF and is further broken down into 5'-phosphoguanylyl-(3'-5')-guanosine (pGpG) by specific phosphodiesterases (PDEs), characterized by the protein domain EAL [2,3,4,5,6]. C-di-GMP is known to bind to I-site of DGCs [7,8], PDEs [9,10], PilZ domain proteins [11,12,13], transcriptional regulators [14,15] and RNA riboswitches [16,17]. Small molecules that could compete with c-di-GMP for binding to receptors have the potential to inhibit biofilm formation and virulence factors production in bacteria. There are several precedents whereby the modifications of natural nucleotides have produced analogues with interesting biological profiles and some of these analogues could even have clinical utility [18,19,20,21,22,23,24,25,26]. Therefore, as a starting point to develop small molecules that could potentially be used to disrupt c-di-GMP signaling, a few groups (including ours) have begun investigating the structure-activity relationship of c-di-GMP binding to both proteins and RNA [27,28,29,30,31,32,33]. Previously, we showed that a conservative change to one of the phosphodiester “bridging” oxygens in c-di-GMP gave an analogue called endo-S-c-di-GMP (**2**, see Figure 1), which has a much lower propensity to form aggregates compared to c-di-GMP [27].

Importantly, endo-S-c-di-GMP could compete with the natural c-di-GMP for binding to RocR, a PDE, but had little affinity for WspR (a DGC) or Alg44 (a PilZ-containing protein). This suggests that modifications to the phosphate moiety of c-di-GMP, at the bridging oxygen position at the phosphodiester moiety could give analogues of c-di-GMP, which could be used to selectively target c-di-GMP receptors. C-di-GMP has also been shown to bind to RNA riboswitches and recent reports suggest that the 2'-modification of c-di-GMP differentially affected binding of these analogues to c-di-GMP riboswitches [17,28,30,33]. We wondered if the simultaneous modification of both the phosphate, at the bridging oxygen position of the phosphodiester moiety, and the 2'-position would lead to another series of c-di-GMP analogues with differential binding profiles. Herein, we describe the synthesis, biophysical characterization and binding to Vc2 RNA of this new class of c-di-GMP analogues, see Figure 1 for structures.

## 2. Results and Discussion

### 2.1. Synthesis of c-di-GMP Analogues

**Scheme 1:**
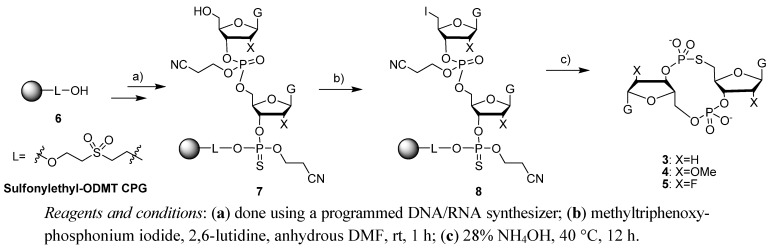
Synthesis of endo-S-c-di-GMP analogues (**2**–**5**).

Endo-S-c-di-GMP analogues **3**–**5** were easily synthesized on a solid support [34], following the strategy outlined in Scheme 1. Briefly, a guanosine phosphoramidite was coupled to a phosphate CPG and the first nucleotide was sulfurized using commercially available Beaucage reagent. The second coupling was done, also with the same guanosine phosphoramidite, but this time iodine was used for the oxidation step to give an interlinking phosphate. The DMT group was then deprotected, using trichloroacetic acid and the 5'-hydroxyl group was converted into an iodide. The dinucleotide was then cleaved from the solid support with NH_4_OH (28% in water). This step also cleaved the cyanoethyl protecting group on the phosphate as well as the acetyl group on the exocyclic amine of the guanine. Upon cleavage from the solid support, a cyclization ensued via a S_N_2 displacement of the 5'-iodide with the 3'-phosphorothioate group. The 2'-OTBS group (in compound **2**) was then deprotected, using NEt_3_**^.^**3HF.

### 2.2. Polymorphism of c-di-GMP Analogues

Polymorphism of c-di-GMP, which depends on physiological conditions (such as the concentration of cations), is relevant to the biological effect of c-di-GMP because different proteins bind to different conformational or aggregation states of c-di-GMP. For example, c-di-GMP binds to most I-sites of DGCs as a self intercalated dimer [7] whereas for PDEs and some PilZ proteins, c-di-GMP binds to the active site as a monomer in either the open form (whereby the two guanines are relatively far away to each other) [9] or the closed form (whereby the two guanines are relatively close to each other) [12]. Recently Chou *et al*. further observed that different proteins could bind to different conformers (with different glycosidic bond angles and/or sugar pucker modes) of the open form of c-di-GMP [35]. Thus it is possible to selectively target c-di-GMP receptors with c-di-GMP-like molecules that mainly exist in a particular conformation, which nicely fits into the binding site of an effector protein or RNA.

C-di-GMP (**1**) at millimolar concentrations and in the presence of potassium cations also forms several polymorphs, including dimers, tetramers and octamers (which contain G-tetrads) [27,36,37]. Interestingly, by replacing one of the bridging oxygens of the phosphodiester units in c-di-GMP with a sulfur atom (a conservative modification) to give an analogue that we name endo-S-c-di-GMP (**2**), the propensity to form G-quadruplex drastically diminishes [27]. We have proposed that one could make a gross prediction for the relative propensity of c-di-GMP analogues to form G-quadruplexes by calculating the relative energies of one of the open conformers (where the two guanines are 13.5 Å apart) to the closed conformer (where the two guanines are 6.8 Å apart) [27]. It would be computationally prohibitive to evaluate all of the energies of all possible conformers of c-di-GMP. To simplify the computational study, the structures computed in this paper were chosen as the closed conformer (*anti* C3'-endo) or one of the three open conformers (*anti* C2'-endo) [35]. Following our precedent [27], and using Gaussian 09 software [38] we obtained relative energy differences between the “closed” and “open” conformers of c-di-GMP and each analogue (**2**–**5**), see Table 1. The closed conformers of both c-di-GMP (**1**), endo-S-c-di-GMP (**2**) and 2'-F-endo-S-c-di-GMP (**5**) seem to be more stable than the open conformers, whereas for 2'-H-endo-S-c-di-GMP (**3**) the energies of both conformers are similar. On the other hand, the open conformer of 2'-OMe-endo-S-c-di-GMP (**4**) appears to be more stable than the closed conformer. We note however that these calculations are simplistic and do not take into account all of the possible c-di-GMP conformers. Additionally this calculation might not have modeled hydration and salt effects very well, so the obtained values have to be interpreted with caution. Nonetheless, the computed data reveals that subtle differences at the phosphate and 2'-position of c-di-GMP could drastically affect the conformation of the molecule (conformational steering). Based on our computational study, we expected that the conformational preference for 2'-F-endo-S-c-di-GMP (**5**) would be similar to endo-S-c-di-GMP (**2**) whereas 2'-H-endo-S-c-di-GMP (**3**) and 2'-OMe-c-di-GMP (**4**) would behave differently from endo-S-c-di-GMP (**2**) and might have a lower propensity to form G-quadruplexes, because the closed conformer, which is required for G-quadruplex formation is predicted to be the minor conformer in these analogues.

**Table 1:** Computed energy difference between “open” and “closed” forms of c-di-GMP (**1**) and analogues (**2**–**5**).

	Δ*E^sol^*(open–closed) *^a^*	ratio (open:closed) *^b^*
c-di-GMP (**1**)	1.9	1:25
endo-S-c-di-GMP (**2**)	1.3	1:9
2'-H-endo-S-c-di-GMP (**3**)	−0.3	2:1
2'-OMe-endo-S-c-di-GMP (**4**)	−2.2	39:1
2'-F-endo-S-c-di-GMP (**5**)	0.5	1:3

*^a^* The electronic energy was computed with Gaussian 09 software with HF/6-31G(d) basis set. Solvent effect (H_2_O) was calculated using Onsager’s model in a self-consistent reaction field; *^b^* The ratio was determined from the equilibrium constant *K*, obtained fromthe equation Δ*E* = −R*T*ln*K* (*T* = 298 K).

When c-di-GMP forms G-quadruplexes, each monomer unit exists in the closed conformer [36]. We wondered if the trend seen in our computational study (Table 1) would correlate with the aggregative properties of the various endo-S-c-di-GMP analogues. We used NMR to characterize the aggregates in the absence or presence of potassium. Previously we used DOSY experiment followed by T_1_/T_2_ relaxation analysis to obtain the diffusion constants of c-di-GMP (**1**) and endo-S-c-di-GMP (**2**) [27]. Because an inverse relationship exists between the size of a particle and diffusion rate, the T_1_/T_2_ relaxation analysis can be used to deduce the size of an aggregate, if data for the monomer can be obtained.

**Figure 2:**
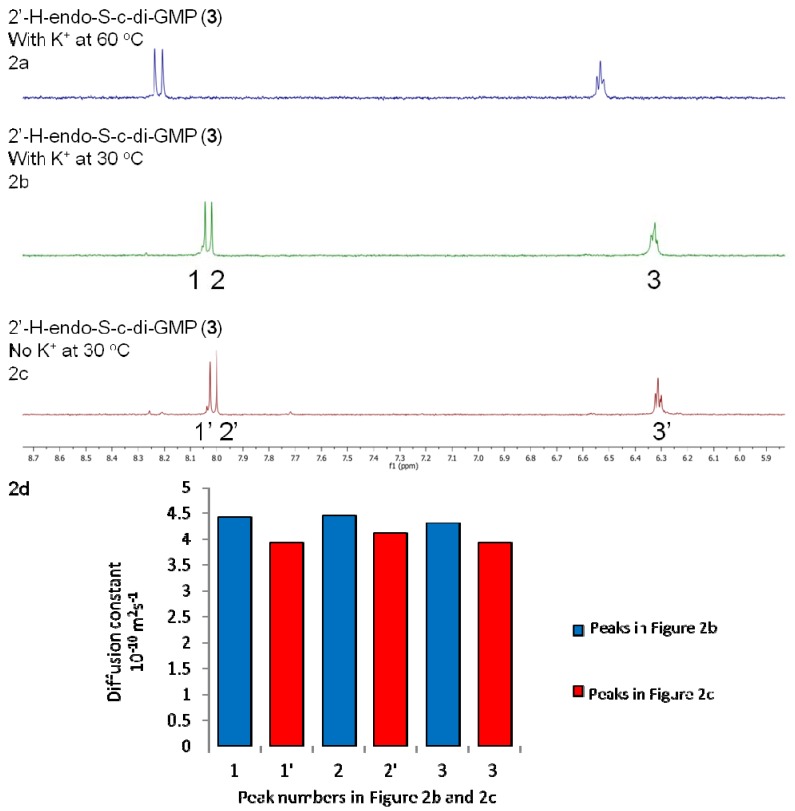
^1^H-NMR stacked spectra of 3.0 mM analogue **3** in D_2_O. (**a**) 3.0 mM 2'-H-endo-S-c-di-GMP, 100 mM KCl, 60 °C. (**b**) 3.0 mM 2'-H-endo-S-c-di-GMP, 100 mM KCl, 30 °C. (**c**) 3.0 mM 2'-H-endo-S-c-di-GMP, no metal cation, 30 °C. (**d**) 3.0 mM 2'-H-endo-S-c-di-GMP, T_1_/T_2_ relaxation analysis (from DOSY experiments).

**Figure 3:**
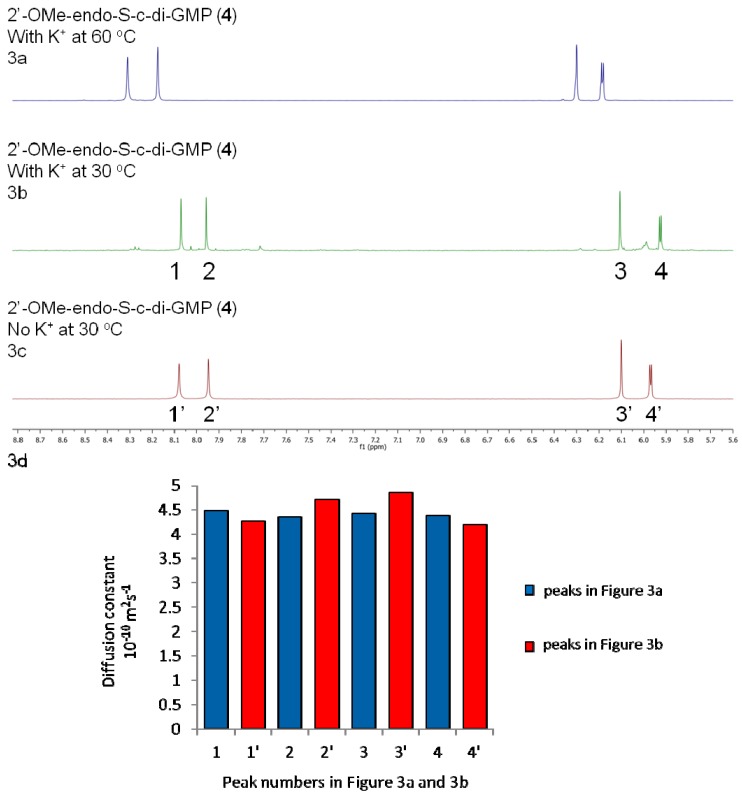
^1^H-NMR stacked spectra of 3.0 mM analogue **4** in D_2_O. (**a**) 3.0 mM 2'-OMe-endo-S-c-di-GMP, 100 mM KCl, 60 °C. (**b**) 3.0 mM 2'-OMe-endo-S-c-di-GMP, 100 mM KCl, 30 °C. (**c**) 3.0 mM 2'-OMe-endo-S-c-di-GMP, no metal cation, 30 °C. (**d**) 3.0 mM 2'-OMe-endo-S-c-di-GMP, T_1_/T_2_ relaxation analysis (from DOSY experiments).

NMR data revealed that the propensities of 2′-H-endo-S-c-di-GMP (**3**) and 2′-OMe-endo-S-c-di-GMP (**4**) to form higher aggregates are lower than for c-di-GMP (**1**) and endo-S-c-di-GMP (**2**) (see Figure 2b and Figure 3b, which show that no new peaks appeared when potassium was added to a solution containing these two endo-S-c-di-GMP analogues).

**Figure 4:**
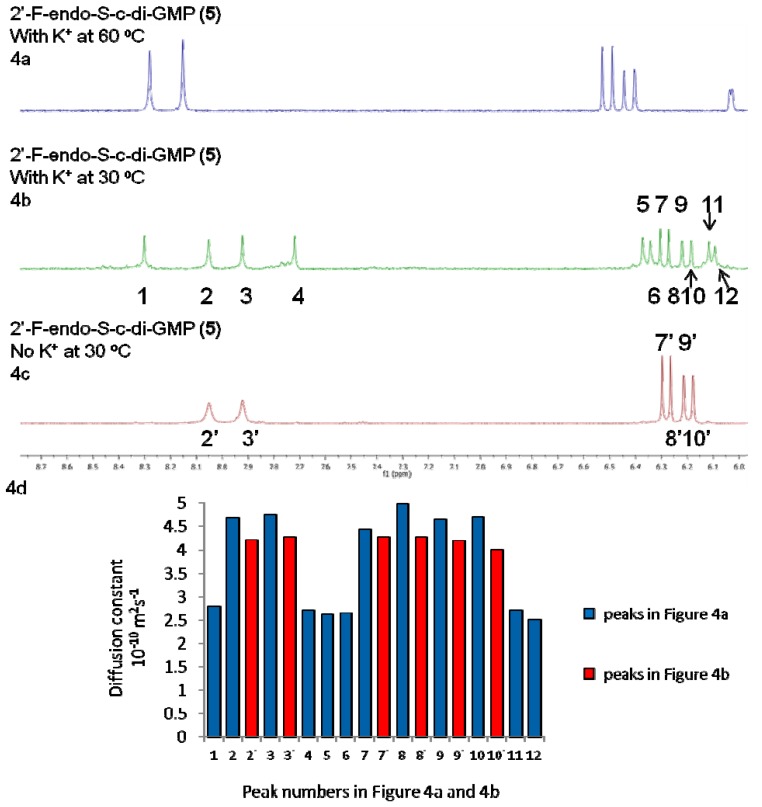
^1^H-NMR stacked spectra of 3.0 mM analogue **5** in D_2_O. (**a**) 3.0 mM 2'-F-endo-S-c-di-GMP, 100 mM KCl, 60 °C. (**b**) 3.0 mM 2'-F-endo-S-c-di-GMP, 100 mM KCl, 30 °C. (**c**) 3.0 mM 2'-F-endo-S-c-di-GMP, no metal cation, 30 °C. (**d**) 3.0 mM 2'-F-endo-S-c-di-GMP, T_1_/T_2_ relaxation analysis (from DOSY experiments).

However, 2'-F-endo-S-c-di-GMP (**5**) formed higher aggregates (octamers) in the presence of potassium cations, see Figure 4. For 2'-F-endo-S-c-di-GMP, the two singlets at 8.05 and 7.92 ppm are assigned to the two guanines H8 and two sets of doublet at 6.28 and 6.18 ppm are assigned to the anomeric H1' (see Figure 4c). We notice that the line width of the 2'-F-endo-S-c-di-GMP peaks at 8.05 and 7.92 ppm are quite broad at 30 °C but become narrow when potassium is added. Grzesiek and co-workers have shown that monomeric c-di-GMP is in equilibrium with dimeric form and it is plausible that the line broadening in the absence of potassium is the effect of the dynamic process rising from the monomer-dimer exchange [39].

An alternative explanation could be that the line broadening is due to conformational heterogeneity (*i.e.*, introduction of a fluorine atom at the 2' position changes the C2'-*endo* or C3'-*endo* equilibrium in the sugar ring, and the addition of K^+^ ion changes this equilibrium rate). Upon adding 100 mM K^+^ to a solution containing 2'-F-endo-S-c-di-GMP (**5**), more peaks appeared near 8.00 ppm and 6.10 ppm, (see Figure 4b), which we interpret as evidence of aggregate formation [27]. Our interpretation is augmented by the fact that heating the solution to 60 °C resulted in the disappearance of the additional peaks, meaning that the new species that gave rise to the additional peaks contained non-covalent interactions. Additionally, DOSY experiments also confirmed that potassium promoted higher aggregation of 2'-F-endo-S-c-di-GMP; the new peaks (8.30 and 7.72 ppm, see peaks **1** and **4** on Figure 4b) that appeared in the 2'-F-endo-S-c-di-GMP ^1^H-NMR, when potassium was added, have diffusion constants of 2.80 × 10^−10^ m^2^s^−1^ and 2.72 × 10^−10^ m^2^s^−1^, whereas the peak at 8.00 and 6.10 ppm (see peaks **2** and **3** on Figure 4b) have diffusion constants 4.67 × 10^−10^ m^2^s^−1^ and 4.75 × 10^−10^ m^2^s^−1^. If peaks **2** and **3** mainly correspond to monomeric c-di-GMP with calculated radius of 6.82 Å, then the peaks at **1** and **4**, which have experimental diffusion constants of 2.80 × 10^−10^ m^2^s^−1^ and 2.72 × 10^−10^ m^2^s^−1^ would correspond to a species of radius 11.60 Å. This radius is similar to the calculated radius (11.92 Å) of octameric 2'-F-endo-S-c-di-GMP and hence provides additional evidence that the addition of potassium to 2'-F-endo-S-c-di-GMP promotes aggregation into octameric forms.

We have previously shown that endo-S-c-di-GMP (**2**) does not readily form G-quadruplexes and commented that c-di-GMP G-quadruplex formation is sensitive to modifications (even conservative ones) [27]. Here we observe that a double modification (phosphate to phosphorothiolates and 2'-OH to 2'-F) can restore the propensity to form higher aggregates, probably G-quadruplexes. Conversely, we found that for the other two double modifications investigated in this study (2'-H and 2'-OMe endo-S analogues), higher aggregates formation was suppressed.

### 2.3. Binding of c-di-GMP and Analogues (2–5) to Vc2 c-di-GMP Riboswitch

**Figure 5:**
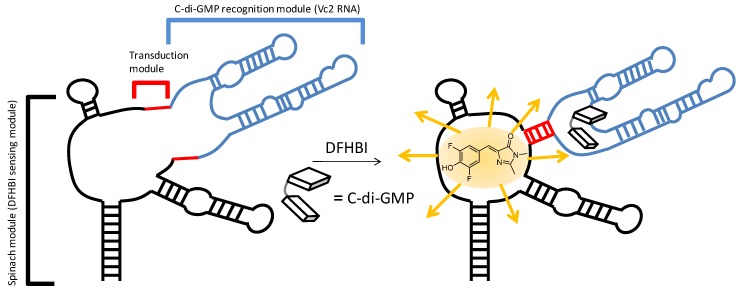
c-di-GMP fluorescent riboswitch. Sequence of RNA is, 5'-GAC GCG ACU GAA UGA AAU GGU GAA GGA CGG GUC CAG GC C GCA CAG GGC AAA CCA UUC GAA AGA GUG GGA CGC AAA GCC UCC GGC CUA AAC UUC GGU AGG UAG CGG GGU UAC CGA GC CUU GUU GAG UAG AGU GUG AGC UCC GUA ACU AGU CGC GUC-3'.

One of the motivations for making c-di-GMP analogues is to obtain small molecules that could have the potential to disrupt c-di-GMP signaling in bacteria. C-di-GMP has been shown to bind to several receptors types (both proteins and RNA) [27,28,29,30,31,32,33]. The crystal structures of c-di-GMP bound to both class I and II riboswitches have been solved [40,41,42]. For class I riboswitch, c-di-GMP is bound in the closed form (see Figure 5).

**Figure 6:**
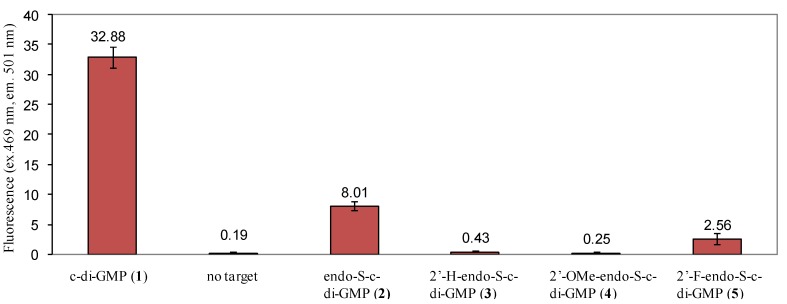
Differential binding of endo-S-c-di-GMP analogues to fluorescent Vc2 RNA riboswitch [43]. Conditions: [RNA, SP_2] = 1 µM, [c-di-GMP and/or analogues] = 10 µM. Buffer: 100 mM HEPES (pH 6.8) containing 100 mM NaCl, 100 mM KCl and 6 mM MgCl_2_.

In a detailed study by Strobel and co-workers, it was demonstrated that substitution of the 2'-position of c-di-GMP was detrimental for binding to class I riboswitch, Vc2 RNA [28]. We have also revealed that the modification of the phosphate moiety of c-di-GMP decreased affinity for class I riboswitch Vc2 RNA [33], whereas binding to RocR (a phosphodiesterase) was not affected [27]. Therefore it might be possible to design c-di-GMP analogues that could selectively bind to one class of c-di-GMP receptor (for example proteins) and not others (for example RNA riboswitches). We investigated if the simultaneous modification of the phosphate and 2'-position of c-di-GMP would additively abrogate binding to the class I riboswitch. Several binding assays to investigate the binding of c-di-GMP and analogues to RNA receptors have been described, including competition with radio-labeled c-di-GMP [28], equilibrium microdialysis [33] and fluorescent c-di-GMP sensor, based on Vc2 RNA riboswitch (class I) [43] To gain a qualitative picture of the relative binding order of analogues, we opted to use the safer but qualitative fluorescent binding assay for c-di-GMP or analogue. The fluorescent c-di-GMP sensor would bind to c-di-GMP or analogue in the riboswitch binding site and the binding event is transduced to another domain, which rearranges to bind to DFHBI, a molecule that is weakly fluorescent in water but becomes highly fluorescent upon binding to RNA (called Spinach RNA). Using this class I riboswitch sensor, it was determined that the relative binding of c-di-GMP and analogues to class I riboswitch is as follows: c-di-GMP (**1**) > endo-S-c-di-GMP (**2**) > 2'-F-endo-S-c-di-GMP (**5**) > 2'-H- and 2'-OMe-endo-S-c-di-GMP (**3** and **4**), see Figure 6. We conclude that the modifications of the phosphate and 2'-position of c-di-GMP are additively detrimental to class I riboswitch binding. Curiously we note that this trend is also observed in the computed open:closed conformer ratio (Table 1). The computed energy differences between the various closed:open forms of c-di-GMP analogues (≤2.2 kcal/mol) is however too small, compared to the binding affinity of c-di-GMP and the riboswitch (>30 kcal/mol) so it is unlikely that the conformer distribution solely accounts for the differences seen in analogue binding to class I riboswitch.

## 3. Experimental

### 3.1. General

Phosphoramidites used in the synthesis of c-di-GMP and analogues were purchased from ChemGenes. DNA/RNA synthesis grade acetonitrile, anhydrous pyridine, triethylamine and other chemicals used in the synthesis were purchased from Sigma-Aldrich (St. Louis, MO, USA) unless noted otherwise. NMR spectra were measured on a Bruker AVANCE II 600 MHz spectrometer and UV absorbance spectra were obtained on a JASCO V-630 spectrophotometer using a 1 cm path length cuvette. The concentration of a stock solution of c-di-GMP and c-di-GMP analogues was determined by the measuring of the absorbance at 253 nm for c-di-GMP and c-di-GMP analogues, using 28,600 M^−1^cm^−1^ as a molar extinction coefficient for all compounds. Mass spectra were measured on a JEOL AccuTOF (Model # JMS-T100CS, Tokyo, Japan) with ESI ion source. Dinucleotides were synthesized by Applied Biosystems 392 DNA/RNA synthesizer.

### 3.2. Synthesis

Sulfonylethyl-ODMT CPG (solid support, 10 µmol/g) was prepared following literature [44]. It was loaded (0.1 g) into the synthesis columns and sealed tightly. A DNA/RNA synthesizer was used for making the dinucleotide. Two cycles of standard 1.0 μmol RNA program were performed with an interruption before the oxidation step during the first cycle. In place of the oxidation step, a sulfurization step was performed using a solution of Beaucage reagent (1 mg/mL in anhydrous acetonitrile, 1 mL) for 30 min. After the reaction was done, the synthesis columns were put back on to the DNA/RNA synthesizer for the rest of the synthesis cycles. The synthesis columns were then washed with anhydrous acetonitrile for another 60 s on the DNA/RNA synthesizer. The CPG beads were collected into round-bottom flask and further dried under the high vacuum for 5 h while gently stirring. Anhydrous DMF (10 mL) was then added to the flask, followed by a rapid transfer of methyltriphenoxyphosphonium iodide (0.95 g, 2.10 mmol) and 2,6-lutidine (1 mL, 8.61 mmol) under argon. The reaction was stirred for 1 h at RT before an aqueous solution of Na_2_S_2_O_3_ (1 M, 30 mL) was poured into the reaction mixture. The CPG beads were washed with H_2_O (10 mL × 3) and methanol (10 mL × 3) and stirred with ammonia (30% NH_4_OH in water, 15 mL) at 40 °C for 12 h. The reaction mixture was then filtered and rinsed with H_2_O (10 mL × 3). The combined filtrate was concentrated and for analogues (**3**–**5**), the crude products were directly subjected to HPLC purification (Nacalai tesque 5C18-PAQ column). HPLC condition: 1 → 11% B, 0 → 16 min (A: 0.1 M TEAA (triethylammonium acetate) in water; B: acetonitrile). The fractions collected from HPLC were concentrated and washed with acetone (2 mL × 5) to remove the excess of TEAA buffer. 3–14 mg products of analogues (**3**–**5**) were collected as white solid (estimated yields of 18–83% were obtained, based on the estimated loading of the CPG beads).

*2'-H-endo-S-c-di-GMP* (**3**). ^1^H-NMR (500 MHz, D_2_O, water suppression, 50 °C) δ 8.26 (s, 1H), 8.23 (s, 1H), 6.65–6.43 (m, 2H), 5.41 (s, 2H), 4.69–4.48 (m, 2H), 4.46–4.27 (m, 2H), 3.33 (dd, *J* = 13.3, 5.7 Hz, 2H), 3.23 (ddd, *J* = 20.6, 13.3, 6.1 Hz, 2H), 3.02 (dt, *J* = 13.6, 6.2 Hz, 1H), 2.92 (ddd, *J* = 11.0, 5.7, 4.9 Hz, 1H).^31^P-NMR (202 MHz, D_2_O, 50 °C) δ 19.7, −0.3. ESI^−^/MS for [C_20_H_23_N_10_O_11_P_2_S]^−^: calculated 673.0749, Found: 673.0702.

*2'-OMe-endo-S-c-di-GMP* (**4**). ^1^H-NMR (500 MHz, D_2_O, 50 °C) δ 8.31 (s, 1H), 8.18 (s, 1H), 6.30 (s, 1H), 6.19 (d, *J* = 4.5 Hz, 1H), 5.35 (dt, *J* = 10.0, 5.0 Hz, 1H), 5.21 (td, *J* = 9.0, 5.0 Hz, 1H), 4.91 (t, *J* = 4.5 Hz, 1H), 4.66–4.52 (m, 4H), 4.39–4.23 (m, 1H), 3.87 (s, 3H), 3.76 (s, 3H), 3.52 (ddd, *J* = 14.0, 11.5, 7.5 Hz, 1H), 3.32 (ddd, *J* = 14.0, 11.5, 3.0 Hz, 1H).^31^P-NMR (202 MHz, D_2_O, 50 °C) δ 18.9, −0.7. ESI^–^/MS for [C_22_H_27_N_10_O_13_P_2_S]^–^: calculated 733.0960, Found: 733.0981.

*2'-F-endo-S-c-di-GMP* (**5**). ^1^H-NMR (500 MHz, D_2_O, water suppression, 50 °C) δ 8.28 (s, 1H), 8.15 (s, 1H), 6.51 (d, *J* = 19.0 Hz, 1H), 6.42 (d, *J* = 19.0 Hz, 1H), 5.98 (dd, *J* = 52.0, 3.5 Hz, 1H), 5.80 (dd, *J* = 51.5, 4.0 Hz, 1H), 5.50 (tdd, *J* = 13.0, 8.5, 5.0 Hz, 1H), 5.35 (dtd, *J* = 22.5, 9.0, 4.0 Hz, 1H), 4.63 (d, *J* = 12.5 Hz, 1H), 4.33 (d, *J* = 12.0 Hz, 1H), 3.69 (dt, *J* = 13.5, 5.0 Hz, 1H), 3.30 (t, *J* = 11.0 Hz, 1H).^31^P-NMR (202 MHz, D_2_O, 50 °C) δ 19.3, −0.5. ESI^–^/MS for [C_20_H_21_F_2_N_10_O_11_P_2_S]^−^: calculated 709.0561, Found: 709.0596.

### 3.3. Sample Preparation for Spectrometric Measurements

C-di-GMP or c-di-GMP analogues, water, and metal solution were mixed, heated to 95 °C and kept at 95 °C for 5 min, cooled back to room temperature for 15 min, then incubated in a refrigerator for 12 h.

### 3.4. DOSY NMR Experiments and T_1_/T_2_ Relaxation Analysis

The Bruker AVANCE II 600 MHz spectrometer was used to determine diffusion constants. Shigemi NMR tubes (D_2_O) were used for all experiments. DOSY was measured with the stimulated echo pulse sequence (Bruker pulse program stebpgp1s19) using bipolar gradient pulses and watergate 3-9-19 to suppress the solvent. Key acquisition parameters for the DOSY experiment include the big delta (*Δ*) at 0.09 s, the number of scans at 32, relaxation delay at 2.5 s, and the gradient strength was varied 32 times linearly from 5 to 95%. The gradient pulse length (small delta δ) within the range of 1.4–1.8 ms was optimized under the experiment conditions until the region of 6.0–9.0 ppm showed good decays for the major peaks. The data were processed with TopSpin 2.1 software with T_1_/T_2_ relaxation analysis. Exponential function was applied for the raw data and the curve-fitting of the decays was based on the area of the peaks.

### 3.5. Sample Preparation of Fluorescence Measurement

C-di-GMP or analogues and DFHBI concentrations were determined via UV absorbance measurements (c-di-GMP at 253 nm and DFHBI at 405 nm) and 28,600 M^−1^cm^−1^ (c-di-GMP), 11,864 M^−1^cm^−1^ (DFHBI) were used as extinction coefficients to calculate concentrations. RNA in buffer solution was heated to 80 °C for 5 min and cooled down to room temperature in 15 min. Then MgCl_2_ and c-di-GMP were added and the sample was kept for 12 h at room temperature.

### 3.6. Fluorescence Measurement

DFHBI was added to samples and fluorescence was monitored by excitation at 496 nm and emission at 501 nm.

## 4. Conclusions

In conclusion, we reveal that by modifying both the phosphate and 2'-positions of c-di-GMP, one could arrive at analogues that show differential biophysical (conformational steering [27]) and biochemical properties. Replacement of one of the oxygen atoms in phosphodiesters can affect both structure and the enzymatic processing of these phosphorothiolate analogues. Usually, it is assumed that the replacement of the exocyclic oxygen with sulfur is less conservative than replacing the endocyclic bridging oxygen. Herein, we reinforce that even a presumed conservative change to the endocyclic oxygen in c-di-GMP can have profound effects in both the property of the nucleotide and binding to receptors [27].
